# Increase of Neutrophil Activation Markers in Venous Thrombosis—Contribution of Circulating Activated Protein C

**DOI:** 10.3390/ijms21165651

**Published:** 2020-08-06

**Authors:** Laura Martos, Julia Oto, Álvaro Fernández-Pardo, Emma Plana, María José Solmoirago, Fernando Cana, David Hervás, Santiago Bonanad, Fernando Ferrando, Francisco España, Silvia Navarro, Pilar Medina

**Affiliations:** 1 Haemostasis, Thrombosis, Arteriosclerosis and Vascular Biology Research Group, Medical Research Institute Hospital La Fe (IIS La Fe), 46026 Valencia, Spain; l.martos.marin@gmail.com (L.M.); juliaotomartinez@gmail.com (J.O.); alvarofernandezpardo@gmail.com (Á.F.-P.); plana_emm@gva.es (E.P.); sol_mjo@gva.es (M.J.S.); fernando_cana@iislafe.es (F.C.); bonanad_san@gva.es (S.B.); ferrando_fer@hotmail.com (F.F.); espanya_fra@gva.es (F.E.); 2 Angiology and Vascular Surgery Service, La Fe University and Polytechnic Hospital, 46026 Valencia, Spain; 3 Data Science, Biostatistics and Bioinformatics Unit, Medical Research Institute Hospital La Fe (IIS La Fe), 46026 Valencia, Spain; bioestadistica@iislafe.es; 4 Thrombosis and Haemostasis Unit, Haematology Service, La Fe University and Polytechnic Hospital, 46026 Valencia, Spain

**Keywords:** neutrophil, venous thrombosis, activated protein C, DNA, cell-free DNA, myeloperoxidase, calprotectin

## Abstract

Upon activation, neutrophils release their content through different mechanisms like degranulation and NETosis, thus prompting thrombosis. The natural anticoagulant activated protein C (APC) inhibits neutrophil NETosis and, consequently, this may lower the levels of neutrophil activation markers in plasma, further diminishing the thrombotic risk exerted by this anticoagulant. We aimed to describe the status of markers of neutrophil activation in plasma of patients with venous thrombosis, their association with the thrombotic risk and the potential contribution of APC. We quantified three markers of neutrophil activation (cell-free DNA, calprotectin, and myeloperoxidase) in 253 patients with venous thromboembolism (VTE) in a stable phase (192 lower extremity VTE and 61 splanchnic vein thrombosis) and in 249 healthy controls. In them, we also quantified plasma APC, soluble endothelial protein C receptor (EPCR), and soluble thrombomodulin (TM), and we genotyped two genetic regulators of APC: the EPCR gene (*PROCR*) haplotypes (H) and the TM gene (*THBD*) c.1418C>T polymorphism. We found a significant increase in plasma cell-free DNA (*p* < 0.0001), calprotectin (*p* = 0.0001) and myeloperoxidase (*p* = 0.005) in VTE patients compared to controls. Furthermore, all three neutrophil activation markers were associated with an increase in the thrombotic risk. Cell-free DNA and calprotectin plasma levels were significantly correlated (Spearman *r* = 0.28; *p* < 0.0001). As expected, the natural anticoagulant APC was significantly decreased in VTE patients (*p* < 0.0001) compared to controls, what was mediated by its genetic regulators *PROCR*-H1, *PROCR*-H3, and *THBD*-c.1418T, and inversely correlated with cell-free DNA levels. This is the largest case-control study that demonstrates the increase in markers of neutrophil activation in vivo in VTE patients and their association with an increased thrombotic risk. This increase could be mediated by low APC levels and its genetic regulators, which could also increase NETosis, further enhancing thrombosis and inflammation.

## 1. Introduction

Blood coagulation is a process that starts quickly after vascular damage in order to repair damaged tissue and maintain blood homeostasis. However, the recent term immunothrombosis [1] has revealed the joint action of the coagulation proteins and the immune cells in the formation of a venous thrombus, although it has only been clarified in vitro. A murine model of deep vein thrombosis (DVT) induced by flow restriction (stenosis) in the inferior vena cava showed the formation of a large clot in which neutrophils constitute the predominant leukocyte subset. Furthermore, neutrophil depletion resulted in a profound inhibition of DVT development [2]. Additionally, neutrophils play an essential role in thrombus resolution, especially in early stages [3]. Upon activation by different stimulus, neutrophils contribute to venous thrombosis by degranulating and releasing neutrophil extracellular traps (NETs) in a process called NETosis [4]. In NETs, released DNA is associated with citrullinated histone 3 along with enzymes such as myeloperoxidase (MPO), calprotectin, and elastase contained within neutrophils [5]. NETs provide a scaffold that stimulates platelet adhesion and aggregation, as well as the formation and deposition of fibrin [6]. In addition, the polyanionic surface of NETs activates the contact phase proteins such as FXII [7], and can also bind and induce the expression of tissue factor to initiate the intrinsic and extrinsic pathways of coagulation, respectively [1,8]. Furthermore, NETs not only activate coagulation but also inhibit the natural anticoagulants. NET-associated elastase and MPO are capable of degrading the tissue factor pathway inhibitor and thrombomodulin (TM) [9,10], thus limiting their anticoagulant function. In addition, histones contained in NETs inhibit the anticoagulant function of activated protein C (APC), by interacting with TM and protein C and inhibiting its activation, further increasing thrombin generation and, with it, the thrombotic risk [11,12]. Besides its anticoagulant role, APC also displays anti-inflammatory and cytoprotective functions [13], which may also be diminished in the presence of NETs, further enhancing the inflammatory scenario.

Moreover, a low level of circulating APC is an independent risk factor for venous thromboembolism (VTE) [14] and early myocardial infarction [15]. APC levels are genetically determined [16] and, in fact, we have previously demonstrated that mutations in the proteins that comprise the quaternary complex needed for protein C activation (thrombin and its receptor TM, and protein C and the endothelial protein C receptor, EPCR) modulate the risk of thrombosis [17,18]. Recent evidence has revealed that APC binds human leukocytes and inhibits NETosis [19]. Therefore, increased NETosis might occur in clinical situations with low APC levels such as VTE [14]. However, the potential regulation on the risk of thrombosis through NETosis exerted by APC in vivo has not been profoundly addressed.

Previous studies carried out to clarify the role of NETs in thrombosis have been mainly conducted in animal models [2,4,6,20,21]. However, there is already incipient evidence about the relationship between NETs and the risk of thrombosis in patients with various prothrombotic and inflammatory disorders [22,23,24,25,26,27,28,29,30,31,32,33,34]. In the particular scenario of VTE, in a case-control study with a very small number of samples, Díaz et al. [26] observed a significant increase in plasma cell-free DNA (cfDNA) concentration in patients with acute DVT. Likewise, Arnalich et al. [30] revealed that patients with acute massive pulmonary embolism (PE) have higher cfDNA levels than patients with sub-massive PE. Additionally, van Montfoort et al. [25] observed that the increase in plasma levels of nucleosomes and elastase-α1-antitrypsin complexes, markers of neutrophil activation, in the acute phase of DVT is associated with a 3-times higher risk of suffering an event. However, the association of the main markers of neutrophil activation and the thrombotic risk was not evaluated.

This is the largest case-control study that demonstrates the increase in markers of neutrophil activation in vivo in VTE patients and where an increase in the thrombotic risk was demonstrated. In addition, these patients have lower APC plasma levels which in turn increase thrombotic risk and may promote NETosis in VTE patients.

## 2. Results

### 2.1. Clinical Characteristics of the Study Subjects

A total of 253 Caucasian VTE patients were enrolled (153 men and 100 women). The clinical characteristics of the study subjects are depicted in Table 1. Of them, 192 patients had lower extremity VTE (106 men and 86 women) and 61 had SVT (47 men and 14 women).

SVT patients were older (median 58 years) than lower extremity VTE patients (45 years) and controls (41 years), and all patients together were older (47 years) than controls (*p* < 0.001 in all cases, Table S1). Additionally, the median age at the first thrombotic event was 42 years in lower extremity VTE patients and 53 years in SVT patients. These disparities in age between both VTE subgroups, together with the interest in exploring the role of neutrophil activation markers in different thrombotic scenarios, led us to analyze lower extremity VTE and SVT independently. A higher proportion of men was present in the SVT group (77%) compared to lower extremity VTE (55%) (*p* < 0.05) and to controls (47%) (*p* < 0.001, Table S1).

Forty-eight lower extremity VTE patients (25%) had spontaneous thrombosis, which is defined as thrombosis in the absence of known triggering factors (use of oral contraceptives, pregnancy, puerperium, surgery, trauma, or immobilization), whereas only 3 SVT patients (5%) had spontaneous thrombosis (*p* < 0.001, Table S1). Regarding blood cell counts, we found lower levels of leukocytes, neutrophils, lymphocytes, and platelets in SVT patients than in lower extremity VTE or controls (*p* < 0.001 in all comparisons, Table S1). We also observed lower levels of lymphocytes (*p* < 0.01) and platelets (*p* < 0.05) in lower extremity VTE patients than in controls. Interestingly, no differences in neutrophil counts were observed between lower extremity VTE and controls. We also evaluated the neutrophil-to-lymphocyte ratio (NLR) among the clinical groups studied, defined as the absolute neutrophil count divided by the absolute lymphocyte count. We found a higher NLR in SVT patients than in lower extremity VTE (*p* < 0.01) and controls (*p* < 0.001). When all patients together were compared to controls, we found lower levels of leukocytes (*p* < 0.001), neutrophils (*p* < 0.01), lymphocytes (*p* < 0.001), platelets (*p* < 0.001), and higher levels of basophils (*p* < 0.05) and NLR (*p* < 0.01) in patients than in controls.

The prevalence of classical prothrombotic polymorphisms, such as factor V Leiden and prothrombin g.20120G>A, was similar to that described in other series (Table 1). A higher proportion of FVL was observed in lower extremity VTE patients (13.5%) and in all patients together (10%) than in controls (2%) (*p* < 0.001 and *p* < 0.01, respectively). A higher proportion of lower extremity VTE patients had recurrent thrombosis (29.7%) whereas only 9.8% of SVT patients experienced recurrences (*p* < 0.01). However, the rate of familial thrombosis was similar between both groups (Table S1). Statistical differences in the complete set of clinical variables registered among the study groups are depicted in Table S1.

### 2.2. Levels of Neutrophil Activation Markers in Patients and Healthy Controls

**Figure 1:**
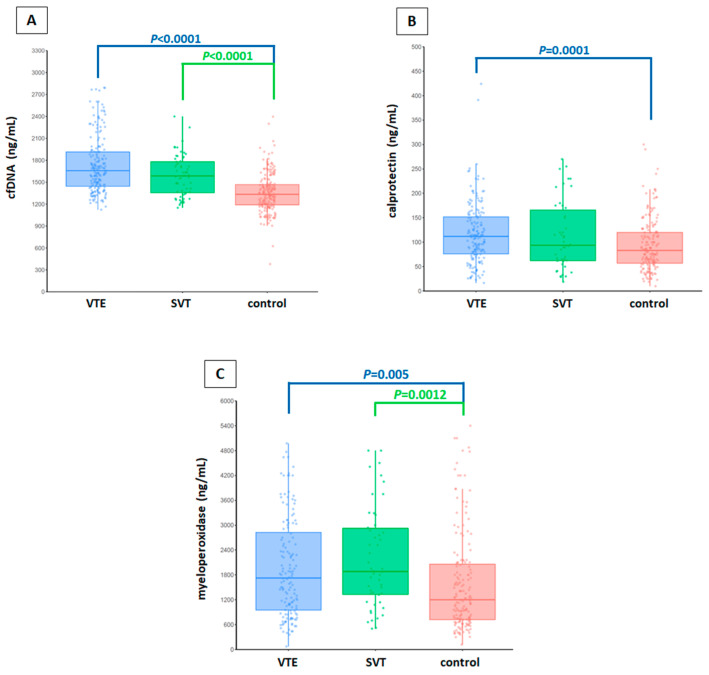
Levels of each neutrophil activation marker in plasma of the clinical groups studied (lower extremity VTE, SVT, and healthy controls). (**A**) cfDNA levels. (**B**) calprotectin levels. (**C**) myeloperoxidase levels. In each graph the median concentration of the marker studied is represented as a dark line. The lower and upper lines of each box represent the first and third quartile, respectively. The length of the whiskers indicates 1.5 interquartile ranges. The observations are represented as circles. Differences in the levels of each neutrophil activation marker between patients and controls were analyzed with linear regression models. All experiments were performed in duplicate. Results were considered statistically significant at *p* < 0.05. All statistical analyses were performed using R (version 3.5.1). VTE, lower extremity venous thromboembolism; SVT, splanchnic vein thrombosis.

Differences in the levels of each neutrophil activation marker between VTE patients and controls were analyzed with a linear regression model. We observed a very significant increase in cfDNA levels in lower extremity VTE patients (median; 25th–75th percentile, 1657.6; 1443.6–1914.1) (*p* < 0.0001) and SVT patients (1586.4; 1348.9–1782.9) (*p* < 0.0001) compared to controls (1320.9; 1185.7–1465.6) (Figure 1A). Similarly, a very significant increase in calprotectin levels was observed in lower extremity VTE patients (112.1; 75.1–152.6) compared to healthy controls (83.0; 55.0–120.0) (*p* = 0.0001) (Figure 1B). Finally, an increase in MPO levels was also observed in lower extremity VTE patients (1728.5; 947.0–2855.0) (*p* = 0.005) and in SVT patients (1882.5; 1235.0–2970.0) (*p* = 0.0012) compared to controls (1250.0; 712.5–2102.0) (Figure 1C).

To evaluate whether the cause of the thrombosis had an influence on the levels of the neutrophil activation markers, we compared their levels between patients with provoked vs. unprovoked DVT. No differences were observed in any of the neutrophil markers studied (Table S2). No differences were observed in neutrophil activation markers according to VTE recurrences (Table S3).

A significant correlation was observed between levels of cfDNA and calprotectin (Spearman *r* = 0.28; *p* < 0.0001) and also between neutrophil count and calprotectin (Spearman *r* = 0.18; *p* = 0.003) which, to some extent, suggests that both markers might have the same cellular origin. No correlation was observed between any of the neutrophil activation markers studied and age (data not shown).

### 2.3. Neutrophil Activation Markers and Risk of Thrombosis

In order to assess the variation of the thrombotic risk according to the concentration of each neutrophil activation marker studied, three multivariable logistic regressions including age, sex, FVL, and PT g.20210G>A as covariates were adjusted. Markers were included in the models after a logarithmic transformation. Adjusted OR for log(cfDNA) was 87.79, with a 95% CI [32.77, 239.69] (Table S4); adjusted OR for log(MPO) was 1.61, with a 95% CI [1.20, 2.18] (Table S5); and adjusted OR for log(calprotectin) was 1.74 with a 95% CI [1.23, 2.49] (Table S6). Additionally, we performed a multivariable logistic regression model including all three markers of neutrophil activation and age, sex, FVL, and PT g.20210G>A as covariates. Adjusted OR for log(cfDNA) was 43.16 (95% CI [13.86, 139.53]) and the adjusted OR for log(MPO) was 1.46 (95% CI [1.03, 2.09]), while calprotectin did not increase the risk of thrombosis in the presence of the other two neutrophil activation markers (Table S7). This effect might be caused by the correlation between plasma cfDNA and calprotectin.

**Figure 2:**
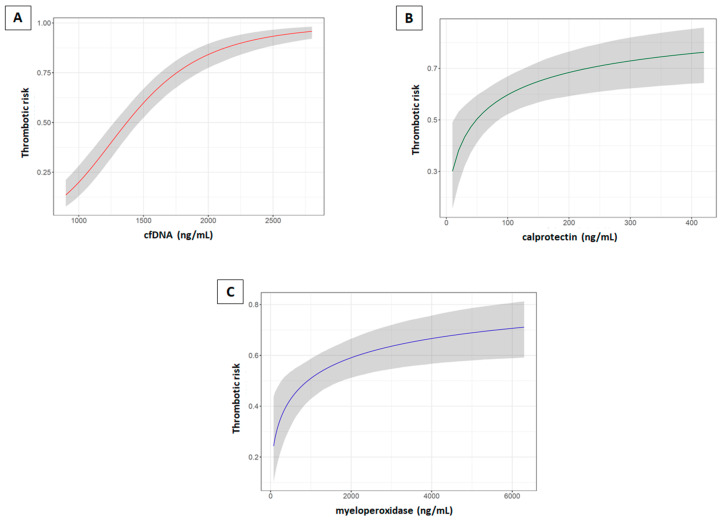
Conditional effects plots that depict the variation of the thrombotic risk according to the values of each neutrophil activation marker studied. (**A**) cfDNA. (**B**) Calprotectin. (**C**) Myeloperoxidase. These conditional effects plots reveal how the risk of VTE varies dynamically with the increase in the levels of each neutrophil activation marker studied, according to the fitted logistic regression models. As a result, the probability of VTE of a given individual is more accurately estimated for every concentration of the marker studied given that the VTE risk is not estimated by distributing the levels of the marker in several ranges, e.g., like quartiles or percentiles. The solid line represents the average and the shaded area represents the 95% CI values.

As depicted in Figure 2A, at low levels of cfDNA the risk of thrombosis was very low, increasing very sharply until reaching a risk near 1 with a plasma cfDNA concentration ≥2500 ng/mL. Regarding calprotectin (Figure 2B) and MPO (Figure 2C), the risk of thrombosis increased following a logarithmic curve, with a higher slope at low values. Furthermore, since slightly higher levels of neutrophils were found in plasma of healthy controls compared to that of patients’ and these may modify the levels of neutrophil activation markers, we repeated the multivariable logistic regressions adjusting by the aforementioned variables and neutrophil counts, and no differences in the ORs were observed (data not shown).

### 2.4. Activated Protein C Plasma Levels and Its Genetic Regulation

Given that one possible mechanism of an increase in NETosis and therefore in plasma neutrophil activation markers is a decrease in APC levels [19], we also quantified the levels of circulating APC in plasma of 98 lower extremity VTE patients and 153 controls from whom the appropriate plasma samples were available. In line with our previous studies [14], circulating APC levels were lower in VTE patients (median; 25th−75th Percentile, 1.05; 0.83–1.23) than in healthy controls (1.25; 1.08–1.60) (*p* < 0.0001) (Table 2). There [14], we described that APC levels <0.69 ng/mL (5th percentile of the control group) increase the risk of VTE by 4.2-fold. Interestingly, in the present study we found 17 VTE patients (17.3%) with APC levels <0.69 ng/mL, whereas we only found 2 controls (1.3%) with APC levels <0.69 ng/mL. A nearly significant inverse correlation was observed between circulating APC and cfDNA levels in controls (*p* = 0.054), the neutrophil activation marker associated with the greatest increase in thrombotic risk. Thus, a lower APC level may be prompting an increase in NETosis and a subsequent increase in cfDNA levels. No correlation was observed between APC levels and calprotectin or MPO (data not shown).

Variations in normal APC levels are, in part, genetically determined by mutations in the receptors located in the endothelial cell surface needed for its activation, i.e., EPCR and TM [16]. Indeed, *PROCR* H1, *PROCR* H3, and *THBD* c.1418C>T modify the expression of these receptors in the endothelial cell surface thus regulating plasma APC levels and, with it, the VTE risk. In particular, *PROCR* H1 reduces the risk of VTE through an increase in APC levels [17]. *PROCR* H3 promote the shedding of EPCR from the cell membrane (increasing plasma sEPCR) thus reducing protein C activation (decreasing plasma APC) and, consequently, increasing the risk of VTE [17]. Finally, *THBD* c.1418C>T promote a decrease in TM shedding from the cell membrane (decreasing plasma sTM) thus increasing protein C activation (increasing plasma APC) and, with it, reducing the risk of VTE [18]. Therefore, we analyzed in our patients the relation between these polymorphisms and the levels of plasma APC, sEPCR, and sTM. We distributed the APC, sEPCR, and sTM levels according to the aforementioned *PROCR* and *THBD* genotypes (Table 2). As expected, APC levels significantly increased with the number of *PROCR* H1 alleles (*p* = 0.0004). sEPCR levels strongly increased when the number of H3 alleles (*p* < 0.0001) and APC levels strongly decreased with the number of H3 alleles (*p* = 0.0008) (Table 2). Finally, sTM levels decreased with the number of *THBD* c.1418T (*p* = 0.0032) and APC levels tended to increase with the number of T alleles, although the number of individuals studied is rather limited due to lack of DNA sample.

As expected, there was no obvious effect of *PROCR* H1 and H3 on sTM levels, and no effect of *THBD* c.1418T on sEPCR levels (Table 2).

To evaluate whether the cause of the thrombosis had an influence on the levels of APC, sEPCR, or sTM, we compared their levels between patients with provoked vs. unprovoked DVT. No differences were observed in APC, sEPCR, or sTM levels (Table S2).

## 3. Discussion

The increasing evidence about the participation of neutrophils in different disorders and the lack of standardization in the markers addressed has prompted the development of specific assays for NETs measurement in plasma with limited success [35]. These assays detect complexes of different NET´s components. However, when used, NETs do not seem to correlate with VTE extent [35]. Other methods, non-applicable for plasma samples, require isolation and in vitro neutrophil activation [36] or have been developed for NETs detection in paraffin-embedded tissue [37]. Thus, to date, no direct and easy method has been validated to measure NETs in plasma. Furthermore, several components of activated neutrophils, but not intact NETs [38], can trigger coagulation and have been associated with an increase in the risk of thrombosis [25,26,28,30] although these studies are rather limited in sample size and/or specific markers addressed. Accordingly, we aimed to further clarify the status of the main markers of neutrophil activation in plasma of VTE patients and their association with the thrombotic risk.

In our study, we observed an increase in the levels of cfDNA, calprotectin and MPO, in plasma of lower extremity VTE and SVT patients. Differences in neutrophil counts could be the source of variation in plasma activation markers; however, no differences in neutrophil counts were observed between lower extremity VTE patients and controls. In addition, a lower level of neutrophils was observed in SVT, what cannot account for the increase in neutrophil activation markers observed. The neutrophil activation markers studied herein could have a different cellular origin other than neutrophils. Calprotectin and MPO could be released by monocytes, macrophages, or eosinophils, but only to a lesser extent. In fact, calprotectin accounts for approximately 60% of total soluble proteins in the cytosolic fraction of neutrophils [39] and, although low levels are found in other phagocytic cells, it is clinically considered to be neutrophil-specific [40] and higher levels in plasma or feces are found in diseases associated with increased neutrophil activity [41,42,43,44,45,46,47,48,49,50,51,52] and NETs [53]. cfDNA could be released into plasma by apoptotic or necrotic cells. Following the strategy addressed in previous studies [25,26,27,28,29,33,34,54,55], we evaluated whether the different markers of neutrophil activation measured in plasma had the same cellular origin by means of their correlation. We found a significant correlation between levels of cfDNA and calprotectin (*r* = 0.28; *p* < 0.0001), similar to that observed in previous plasma studies [27,33], and also between neutrophil count and calprotectin (Spearman *r* = 0.18; *p* = 0.003) indicating that they might have, to some extent, the same origin, probably an increased activation of neutrophils or NETosis. Additionally, the inflammatory marker NLR has been previously ascertained as a simple and valuable marker for VTE prognosis [56,57,58,59] and also for other thrombotic disorders [60,61,62,63]. Thus, we also evaluated the NLR in our clinical groups. In line with these previous studies, we found a significantly increased NLR in SVT patients and also in all patients when analyzed together compared to healthy controls. In contrast to previous studies, no differences in NLR arose between lower extremity VTE patients and controls and NLR values in our patients were lower than those previously reported, probably because most of these studies were conducted in the acute phase of the disease when an exacerbated inflammatory state may be occurring. Remarkably, SVT patients had a significantly higher NLR than lower extremity VTE patients, what might be influenced by the differences in blood counts in these patients compared to the other two study groups analyzed. Future studies prospectively conducted in larger cohorts of SVT and lower extremity VTE patients might confirm the discrepant NLR in these thrombotic scenarios and its diagnostic value.

VTE is a serious disorder with an incidence of 1.4 per 1000 individuals/year, 20% of which die within a year [64]. The causes of thrombosis are both genetic and acquired; however, despite the large increase in the number of identified risk factors, still a substantial number of thrombotic events occur spontaneously without an apparent origin, which may reflect causes not yet discovered. Provided the interplay between immunity and thrombosis, where neutrophils play a predominant role; markers of neutrophil activation may allow identifying patients with high thrombotic risk, as we previously demonstrated in cancer patients [33,34]. Thus, we explored the relation of neutrophil activation markers and the risk of thrombosis. We found that increased plasma levels of cfDNA, calprotectin and MPO were all associated with a significant increase in the risk of VTE. Age, sex, FVL, PT g.20210G>A or neutrophil count did not modify the risk of thrombosis. However, when all markers of neutrophil activation were analyzed together, only cfDNA and MPO remained significant, what may be caused by the correlation between plasma cfDNA and calprotectin. In line with previous studies, cfDNA was the parameter associated with the greatest increase in the thrombotic risk. Recently, Jiménez-Alcázar et al. [35] highlighted new aspects of the role of cfDNA in the thrombotic risk. In their study, plasma cfDNA levels correlated with VTE extent and predicted PE-related and all-cause mortality in patients with acute VTE aged ≥65 years during a 3-year follow-up. Similarly, Arnalich et al. [30] suggested that cell-free plasma mitochondrial DNA may be a promising marker for predicting 15-day mortality in patients with acute massive PE. DNA seems to be an essential component of activated neutrophils in driving their prothrombotic activities [38]. In fact, previous studies demonstrated that the effective resolution of a clot during thrombolysis not only required fibrin and von Willebrand Factor degradation, but also required the complete lysis of the scaffold structure that composes NETs, mainly DNA [4,65,66]. Moreover, the reduction of DNase I activity in plasma was associated with a higher rate of acute thrombotic microangiopathies [24] and also lupus-associated nephritis caused by the development of autoantibodies against DNA, histones, and neutrophil proteins when NETs were not rapidly eliminated [22]. Remarkably, the negative effects of poorly degraded NETs in these patients could be restored by supplementing plasma with recombinant human DNase I. All in all, our results suggest that once confirmed in a large independent cohort of VTE patients, cfDNA, calprotectin, and MPO might become novel VTE biomarkers and their dysregulation might explicate a subset of unexplained VTE events. Additionally, novel therapeutic strategies might be developed to restore their dysregulation.

Besides its anticoagulant function, APC is able to degrade neutrophil histones and reduce their cytotoxicity, thus decreasing thrombosis, endothelial dysfunction, renal dysfunction, intra-alveolar hemorrhages, organ failure, and death in sepsis models [67,68]. Furthermore, recent evidence has revealed that APC binds human leukocytes and inhibits NETosis in an EPCR-, PAR3-, and Mac-1-dependent manner, what may represent a new anti-inflammatory mechanism of APC [19]. Provided that a low plasma level of APC is an independent risk factor for VTE [14], increased NETosis and reduced histone degradation may also occur in patients with low APC levels such as VTE [14], myocardial infarction [15], and other inflammatory processes [69]. Herein, we evaluated the relation between circulating APC levels and NETosis (through the quantification of the neutrophil activation markers), and we observed significant lower levels of plasma circulating APC in VTE patients than in healthy controls and an increase in neutrophil activation markers. Furthermore, an inverse correlation was observed between circulating APC and cfDNA levels, the neutrophil activation marker associated with the greatest increase in thrombotic risk. Our findings reinforce the hypothesis of a prothrombotic scenario in VTE patients facilitated both by low anticoagulant APC levels and an increase in neutrophil activation markers. Furthermore, these low APC levels would further enhance NETosis and, with it, thrombosis. However, we are aware that our results cannot establish any relation of direct causality between APC and NETosis in VTE patients, what may require additional experiments. Moreover, APC levels are genetically regulated by mutations in the receptors on the endothelial cell surface needed for its activation, i.e., EPCR and TM. *PROCR* H1 and *THBD* c.1418T increase APC levels through an increase in the membrane-bound receptors (decreasing sTM levels) thus leading to an increased protein C activation and, with it, to a decrease in the risk of VTE [17,18]. Contrarily, *PROCR* H3 decrease APC levels by inducing the shedding of the receptor from the endothelial cell membrane (increasing sEPCR levels) thus reducing protein C activation and, with it, increasing the risk of VTE [17]. Therefore, we also evaluated the genetic regulation of APC levels by these polymorphisms in our VTE patients and we confirmed our previous findings. This reduction in APC levels observed in our VTE patients would both increase the risk of thrombosis and may promote NETosis, with the subsequent increase in the neutrophil activation markers observed in these patients. Furthermore, the inhibition of NETosis by APC is EPCR-dependent [19] and we have demonstrated that *PROCR* H1 is anti-thrombotic via an increase in functional membrane-bound EPCR and that *PROCR* H3 is pro-thrombotic via the induction of the shedding of the EPCR from the endothelial cell membrane, which further reinforces this feedback mechanism. Moreover, the reduction in APC levels observed in our patients would also reduce the cytoprotective properties of APC what may contribute, in turn, to maintain a basal systemic chronic inflammatory state that would promote both thrombosis and NETosis.

A limitation of our study is that, contrarily to that observed in previous studies conducted in the acute phase of the disease [25,28,35], we could not reliably detect nucleosomes in plasma given the extremely low levels measured. This effect cannot be related to technical problems since we successfully performed this assay in plasma of cancer and cancer-VTE patients [33,34]. Therefore, we believe that nucleosomes could not be reliably measured probably because our samples were not collected in the acute phase of the thrombotic event or due to pre-analytical differences among studies. Another limitation is that, although patients were studied at least 6 months after the acute event, we cannot know whether the increased levels of the neutrophil activation markers studied are a persistent endogenous characteristic of VTE patients that contributed to the prior thrombotic event or whether they are the consequence of the thrombosis. However, although the half-life of the neutrophil activation markers has not been well-established, a basal NETosis may be occurring provided that these markers are present in plasma of healthy controls. Prospective population-based studies would validate the association of increased plasma levels of these markers and the risk of thrombosis and would ascertain whether their dysregulation explains a portion of current VTE events of current uncertain origin. However, such studies are very hard to conduct given the great sample size and long follow-up required to show significant differences. In turn, many studies of biological markers related to thrombosis have been conducted after the VTE event and in the stable phase of the disease [14,17,70,71,72]. Additionally, APC studies cannot be performed in the acute phase of thrombosis since APC is being consumed, thus impeding the achievement of this study in the acute phase of thrombosis. Current population-based studies cannot be employed to corroborate our results since a special plasma sample is required for APC quantification [14] and because limited or no plasma samples are often available. Finally, other markers of neutrophil activation such as MPO-DNA complexes might be more specific markers of NETosis [73,74] and may shed light on the underlying mechanism responsible for the contribution of neutrophils to thrombosis.

## 4. Materials and Methods

### 4.1. Study Subjects

Our study included 253 unrelated Caucasian patients with at least one objectively confirmed episode of thrombosis who consecutively and prospectively entered the anticoagulation clinic at La Fe University and Polytechnic Hospital (Valencia, Spain) for thrombophilia study, between 2010 and 2015. Objective diagnoses of lower extremity DVT and PE were made by clinical probability, D-dimer levels, compression ultrasonography, ventilation perfusion lung scan and, when necessary, phlebography or pulmonary angiography. Splanchnic vein thrombosis (SVT), another type of DVT, was objectively diagnosed by abdominal computed tomography-scan or ultrasonography. Spontaneous thrombosis was defined as a thrombotic event without known precipitating risk factors (use of oral contraceptives, pregnancy, puerperium, surgery, trauma, or immobilization). Patients under antithrombotic therapy, protein C deficiency, protein S deficiency, antithrombin deficiency, or with known cancer were excluded.

The control group included 249 unrelated Caucasian healthy subjects with no history of thromboembolic disease. Controls were randomly selected to match cases by age, gender, and geographic distribution.

All subjects participated after giving written informed consent according to protocols approved by the ethics review board at La Fe University and Polytechnic Hospital. The study was performed according to the declaration of Helsinki, as amended in Edinburgh in 2000.

### 4.2. Blood Collection

Blood was collected at least 6 months after the thrombotic event (median 1 year), i.e., in the stable phase of the disease. For the quantification of neutrophil activation markers, blood was collected in 4.5 mL Vacutainer tubes (BD Diagnostics, Franklin Lakes, NJ, USA) containing 0.5 mL of 0.109 M trisodium citrate. For the measurement of circulating APC, blood was collected two tubes containing 0.109 M trisodium citrate. Immediately after blood sampling (within 10 s), 46 μL of a mixture of 0.58 M benzamidine-HCl (Sigma Aldrich, Saint Louis, MO, USA) and 0.5 mM D-phenylalanyl-L-prolyl-L-arginine chloromethyl ketone·2HCl (PPACK, Calbiochem, Darmstadt Germany) was added to one citrate tube to inhibit circulating APC, and 46 μL of 1000 U/mL heparin (Rovi, Madrid, Spain) was added to the other citrate tube and the mixture was incubated at 37 °C for 30 min to force APC to complex to its major inhibitor, the protein C inhibitor (PCI) [14]. Plasma was obtained by centrifugation at 1.811× *g* for 30 min at 4 °C and stored in aliquots at −80 °C until further use. For DNA studies, blood was collected in tubes containing K3EDTA.

### 4.3. Quantification of Neutrophil Activation Markers

Markers of neutrophil activation were measured following the strategy addressed previously [25,26,27,28,29,33,34,54,55], i.e., measuring cfDNA and nucleosomes as markers of the nuclear content of neutrophils released upon NETosis, calprotectin as a marker of cytoplasmic content, and myeloperoxidase as a marker of the content of neutrophil granules, both released upon neutrophil activation by different mechanisms. All the experiments were performed in duplicate.

Plasma cfDNA was quantified using the Quant-iTTM PicoGreen dsDNA Kit (Life Technologies, Eugene, OR, USA). Plasma was diluted 1/40 in TE buffer (10 mM Tris-HCl, 1 mM EDTA, pH 7.5). One hundred microliters of diluted plasma was mixed with 100 µL of TE containing Quant-iT™ PicoGreen (200-fold dilution) to label cfDNA. Fluorescence was recorded in a SpectraMax Gemini XS fluorometer (Molecular Devices, San José, CA, USA) at 480 nm excitation and 520 nm emission. cfDNA concentrations were calculated based on a standard curve of known concentrations of λ DNA (Life Technologies) included in each experimental plate. The intra- and inter-assay coefficients of variation were less than 7%.

Nucleosomes are a complex of DNA and histones and represent the basic building blocks of chromatin. A nucleosome comprises about 150–200 bp of DNA wrapped around a core of double represented histone proteins H2A, H2B, H3, and H4. A linker DNA of 10 to 100 bp connects nucleosomes and histone H1 binds to the linker DNA and is important for chromatin organization. Plasma nucleosomes were quantified using the Cell Death Detection ELISA^PLUS^ kit (Roche, Mannheim, Germany) following manufacturer’s instructions. Briefly, nucleosomes were quantified in 20 µL undiluted plasma using a capturing antibody against an epitope shared by all histones and a detecting antibody against DNA. Absorbance at 405 nm (reference wavelength 490 nm) was recorded in a MRX TC Revelation plate reader (Thermo Labsystems, Madrid, Spain). However, levels of nucleosomes in these individuals were extremely low: in a subset of 79 patients and 81 healthy controls the average optical density obtained was 0.08 and 0.07, respectively, near the blank values. Therefore, we discontinued the quantification of nucleosomes in our study.

MPO is a heme protein stored in granules of neutrophils and monocytes known to produce reactive oxygen species. MPO is particularly abundant in neutrophils, accounts for 25% of granular proteins and for 5% of all proteins, and is released together with DNA during NETosis. Plasma MPO was quantified from 1/50 diluted plasma using the MPO (human) ELISA Kit (Abnova, Taoyuan, Taiwan) following manufacturer’s instructions. Absorbance at 450 nm (reference wavelength 620 nm) was recorded in a MRXTC Revelation plate reader (Thermo Labsystems). The intra- and inter-assay coefficients of variation were less than 11%.

Calprotectin (also known as S100A8/A9 heterocomplexes), belongs to a group of calcium-binding S100 proteins that is mainly contained within circulating neutrophils, in which it accounts for 60% of the cytosolic proteins, and it can be found at low concentrations in monocytes and macrophages [39]. Calprotectin is used as a diagnostic marker of inflammation in human plasma [41,46,50,51,52], and is released together with DNA during NETosis [5]. Plasma calprotectin was quantified from 1/50 diluted plasma using the Human Calprotectin ELISA Kit (Hycult Biotech, Uden, TheNetherlands)) following manufacturer´s instructions. Absorbance at 450 nm (reference wavelength 620 nm) was recorded in a MRXTC Revelation plate reader (Thermo Labsystems). The intra- and inter-assay coefficients of variation were less than 10%.

### 4.4. Quantification of Plasma APC, sEPCR, and sTM

Plasma levels of circulating APC were measured by quantifying the concentration of APC:PCI complexes from 1/6 diluted plasma by a sandwich ELISA developed by our group as previously reported [14]. Briefly, microplates were coated with a monoclonal antibody to protein C (PC) and complexes were detected with peroxidase-labeled polyclonal antibodies to PCI. APC concentration in the complex is expressed in nM, assuming a molecular weight of APC of 57,000. Standard curves were constructed with known amounts of in vitro preformed complexes. The detection limit of the assay in plasma was 0.0017 nM (0.1 μg/L) APC. The concentration of APC:PCI in the heparin tube is the sum of the in vivo circulating APC:PCI complexes and the complexes formed in vitro from circulating APC, whereas the concentration of APC:PCI in the benzamidine-PPACK tube reflects the concentration of the in vivo circulating APC:PCI complexes. Hence, the concentration of circulating APC was calculated from the difference in APC:PCI concentration measured in the two tubes. All samples were measured in duplicate. Absorbance at 490 nm (reference wavelength 620 nm) was recorded in a MRXTC Revelation plate reader (Thermo Labsystems). The intra- and inter-assay coefficients of variation were less than 15%.

Plasma levels of soluble EPCR (sEPCR) were measured from 1/51 diluted plasma with the Asserachrom sEPCR ELISA kit (Diagnostica Stago, Asnières sur Seine Cedex, France) [17] following manufacturer’s instructions without additional modifications. All samples were measured in duplicate. Absorbance at 450 nm (reference wavelength 620 nm) was recorded in a MRXTC Revelation plate reader (Thermo Labsystems). The intra- and inter-assay coefficients of variation were less than 7%.

Plasma levels of soluble TM (sTM) were measured from 1/4 diluted plasma with the Imubind Thrombomodulin ELISA kit (American Diagnostica, Burlington, MA, USA) [18] following manufacturer’s instructions without additional modifications. All samples were measured in duplicate. Absorbance at 490 nm (reference wavelength 620 nm) was recorded in a MRXTC Revelation plate reader (Thermo Labsystems). The intra- and inter-assay coefficients of variation were less than 6%.

### 4.5. Genotyping of the Genetic Regulators of Plasma APC: PROCR Haplotypes and THBD c.1418C>T Polymorphism

Genomic DNA of patients and controls was isolated from 200 µL K3EDTA blood using the Wizard^®^ Genomic DNA Purification Kit (Promega, Madison, WI, USA), following the manufacturer’s instructions.

The haplotypes (H) of the gene encoding for EPCR (*PROCR*) H1 and H3 are tagged by the SNPs g.4678G>C (rs9574) and g.4600A>G (rs867186), respectively. Both SNPs are located in *PROCR* exon 4, which includes the 3-UTR of *PROCR*, and it was amplified by using the following primers: forward 5′-GCTTCAGTCAGTTGGTAAAC-3′, and reverse 5′-TCTGGCTTCACAGTGAGCTG-3′ (Gibco BRL, Life Technologies). The 50 µL reaction mixture contained 3 μL of 7 ng/μL DNA, 1 μL of dNTPs (10 mM/each; PCR Nucleotide Mix, Promega), 0.3 μL of 10 pmol/μL forward primer, 0.135 μL of 1485 ng/μL reverse primer, 10% dimethylsulfoxide (Sigma), 5 μL of 1 mg/mL BSA (New England Biolabs, Ipswich, MA, USA), 0.25 μL of 5 U/μL Taq DNA polymerase (Promega), and 5 μL of 670 mM Tris-HCl pH 8.8, 67 mM MgCl_2_, 67 μM EDTA, 166 mM (NH_4_)_2_SO_4_ and 100 mM β-mercaptoethanol (Sigma). The reaction mixture was incubated at 94 °C for 4 min, followed by 32 cycles of 94 °C for 45 s, 55 °C for 45 s and 72 °C for 45 s, with a final extension of 4 min at 72 °C [75]. The g.4600A>G SNP was genotyped by SSCP: 10 μL of amplified PCR product was mixed with 10 μL of 98.75% formamide containing 5 mM EDTA, 0.125% bromophenol blue and 0.125% xylene cyanol (Sigma). The mixture was denatured at 95 °C for 7 min and applied to an 8% polyacrylamide gel containing 7% glycerol, 89 mM Tris-borate, and 2 mM EDTA pH 8.0. Electrophoresis was performed at 125 V for approximately 19 h at a constant temperature of 24 °C (DCODE Universal Mutation Detection System, Bio-Rad Laboratories, Hercules, CA, USA). The gel was silver stained following standard procedure. Controls with the 4600AA, AG, and GG genotypes were included in each gel. When SSCP analysis revealed a shift in the migration pattern of the PCR products, PCR products were directly sequenced to confirm the g.4600AG genotype. Sequencing was performed using CEQ 2000 Dye Terminator Cycle Sequencing Kit and CEQ 2000 DNA Analysis System (Beckman Coulter, Brea, CA, USA). The g.4678G>C was genotyped by restriction analysis with DdeI (5´C/TNAG 3′). Restriction analysis was performed in a 24 μL reaction mixture containing 16 μL of the aforementioned PCR product, 0.2 μL of 10 U/μL DdeI (Gibco BRL, Life Technologies), 2.4 μL of a buffer containing 50 mM Tris-HCl pH 8.0, 10 mM MgCl_2_ and 100 mM NaCl (Gibco BRL, Life Technologies) and 5.4 μL of dH_2_O. The mixture was incubated at 37 °C for 3 h and digestion products were electrophoresed on a 2.5% agarose gel (Agarose MS8 Pronadisa, Hispanlab, Madrid, Spain) containing 0.05 mg ethidium bromide/mL gel and visualized under UV light. In the presence of the mutation (C allele), the original 314 bp fragment is converted in two fragments of 252 and 62 bp. The presence of the 4678CC genotype was confirmed by PCR amplification and direct sequencing in all cases. In addition, about 30% of samples genotyped by restriction analysis as GG or GC were sequenced and, in all cases, the genotype was confirmed.

The c.1418C>T polymorphism (rs1042579) of the gene encoding for TM (*THBD*) was genotyped by direct sequencing with the ABI PRISM^®^ 3730 DNA Analyzer (Applied Biosystems, Foster City, CA, USA), using the following set of primers: forward 5′-GTGGCTTCGAGTGCCACTGC-3′ and reverse 5′-CGCACTTGTACTCCATCTTGGCCCTG-3′ (Gibco BRL, Life Technologies). The reaction mixture contained 3 μL of 7 ng/μL DNA, 10 μL of 5X colorless Go Taq^®^Flexi buffer (Promega), 3 μL of 25 mM MgCl_2_, 1 μL of dNTPs (10 mM/each), 0.3 μL of 10 pmol/μL forward primer, 0.135 μL of 1485 ng/μL reverse primer, 0.25 μL of 5 U/μL Taq DNA polymerase (Promega) and 40.27 μL of dH_2_O. The reaction mixture was incubated at 95 °C for 4 min, followed by 33 cycles of 95 °C for 45 s, 66 °C for 45 s, and 72 °C for 45 s, with a final extension of 4 min at 72 °C [18].

### 4.6. Statistical Analysis

Data were summarized using median (first, third quartile) in the case of continuous variables and relative and absolute frequencies in the case of categorical variables. Differences in the levels of each neutrophil activation marker between clinical groups were analyzed with linear regression models. The associations between the different markers were assessed using Spearman’s correlation. The association between the thrombotic risk and the concentration of each neutrophil activation marker was assessed by adjusting three Bayesian logistic regression models with weakly informative priors for the coefficients, N(0, 1). Additionally, as a sensitivity analysis, a Bayesian logistic regression model was adjusted including all the neutrophil activation markers. Age, sex, FVL, and PT g.20120G>A were included as a cofounder in these models. The association of APC, sEPCR, and sTM with VTE were assessed comparing the levels of each marker between the two clinical groups using the Wilcoxon–Mann–Whitney test and differences within the groups were assessed using the Kruskal–Wallis test. Results were considered statistically significant at *p* < 0.05. All statistical analyses were performed using R (version 3.5.1).

## 5. Conclusions

In conclusion, this is the largest case-control study that demonstrates the increase in markers of neutrophil activation in vivo in VTE patients and where an increase in the thrombotic risk was demonstrated. Furthermore, these patients have lower APC plasma levels what, in turn, increase the thrombotic risk, might promote NETosis and might induce a basal pro-inflammatory state that would further enhance both thrombosis and NETosis. The deep knowledge of the molecular mechanisms triggered by neutrophil components released upon activation will promote strategies to regulate their pro-thrombotic potential.

## Figures and Tables

**Table 1:** Clinical characteristics of the study subjects

Clinical Characteristics	Patients			Controls
	Lower Extremity VTE	SVT	Total	
N (% of total)	192 (68.6)	61 (21.8)	253	249
Age, y	45 (36, 55)	58 (46, 66)	47 (37, 60)	41 (32, 55)
Age at first onset, y	42 (33, 51)	53 (38, 61)	42 (34, 53)	----
Male sex, N (%)	106 (55.2)	47 (77.0)	153 (60.5)	117 (47.0)
Recurrent thrombosis, N (%)	57 (29.7)	6 (9.8)	63 (24.9)	----
Familial thrombosis, N (%)	69 (35.9)	20 (32.8)	89 (35.2)	---
Spontaneous thrombosis, N (%)	48 (25.0)	3 (4.9)	51 (20.2)	----
Leukocytes, × 10^9^/L	6.4 (5.3, 7.4)	4.6 (3.6, 5.5)	5.9 (4.8, 7.1)	6.4 (5.5, 7.9)
Neutrophils, × 10^9^/L	3.5 (2.9, 4.3)	2.6 (2.1, 3.3)	3.2 (2.5, 4.1)	3.7 (3.0, 4.6)
Monocytes, × 10^9^/L	0.5 (0.4, 0.6)	0.5 (0.4, 0.7)	0.5 (0.4, 0.6)	0.5 (0.4, 0.6)
Eosinophils, × 10^9^/L	0.15 (0.10, 0.22)	0.13 (0.08, 0.24)	0.15 (0.10, 0.23)	0.18 (0.10, 0.21)
Basophils, × 10^9^/L	0.01 (0.00, 0.03)	0.01 (0.00, 0.02)	0.01 (0.00, 0.03)	0 (0.00, 0.02)
Lymphocytes, × 10^9^/L	1.90 (1.53, 2.34)	1.12 (0.78, 1.53)	1.71 (1.23, 2.20)	2.10 (1.71, 2.60)
Platelets, × 10^9^/L	223 (188, 257)	97 (67, 153)	205 (143, 248)	238 (204, 276)
Neutrophil-to- lymphocyte ratio	1.81 (1.44, 2.35)	2.26 (1.64, 3.41)	1.90 (1.46, 2.52)	1.68 (1.33, 2.24)
FV Leiden, N (%)				
-/-	166 (86.5)	59 (96.7)	225 (88.9)	244 (98.0)
+/- & +/+	25 & 1 (13.5)	2 & 0 (3.3)	27 & 1 (10.0)	4 & 1 (2.0)
PT g.20210G>A, N (%)				
GG	176 (91.7)	57 (93.4)	233 (92.1)	236 (94.8)
GA & AA	16 & 0 (8.9)	4 & 0 (6.6)	20 & 0 (7.9)	13 & 0 (5.2)

Continuous variables are displayed as median and interquartile range. Categorical variables are displayed as count and percentage.

**Table 2:** Levels of APC, sEPCR, and sTM (median and 25th−75th percentile) in lower extremity VTE patients and controls according to *PROCR* haplotypes and the *THBD* c.1418C>T polymorphism. The association of APC, sEPCR, and sTM with VTE were assessed comparing the levels of each marker between the two clinical groups using the Wilcoxon–Mann–Whitney test and differences within the groups were assessed using the Kruskal–Wallis test. All samples were measured in duplicate. Results were considered statistically significant at *p* < 0.05. All statistical analyses were performed using R (version 3.5.1).

Genotype	APC (ng/mL)					sEPCR (ng/mL)					sTM (ng/mL)				
	n	Patients	n	Controls	M-W Test *p*	n	Patients	n	Controls	M-W Test *p*	n	Patients	n	Controls	M-W Test *p*
All	98	1.05 (0.83–1.23)	153	1.25 (1.08–1.60)	<0.0001	94	102.0 (84.0–122.0)	153	100.0 (82.0–130.0)	0.7720	53	4.16 (3.69–4.73)	129	3.77 (3.33–4.44)	0.0205

HxHx	20	0.91 (0.68–1.07)	32	1.10 (0.90–1.53)	0.0229	19	80.0 (70.0–100.0)	32	87.0 (73.5–106.5)	0.4770	12	4.17 (3.62–4.55)	28	3.92 (3.50–4.70)	0.7455
HxH1	40	1.09 (0.95–1.23)	60	1.26 (1.15–1.52)	<0.0001	38	94.5 (85.5–112.0)	60	96.0 (81.0–112.5)	0.8354	25	4.08 (3.37–4.42)	52	3.76 (3.23–4.41)	0.2534
H1H1	20	1.23 (1.15–1.44)	32	1.31 (1.11–1.72)	0.5472	20	100.5 (86.5–116.0)	32	90.5 (80.5–111.5)	0.4806	8	4.29 (3.95–4.84)	26	3.72 3.07–4.34)	0.0490
K-W test *p*		0.0004		0.0482			0.0171		0.2222			0.5129		0.4800	

H1H3	7	0.97 (0.89–1.03)	20	1.29 (1.10–1.72)	0.0026	6	260.0 (234.0–294.0)	20	251.5 (208.5–281.0)	0.5838	5	5.90 (3.59–6.25)	17	3.84 (3.43–4.54)	0.1367

HxHx	20	0.91 (0.68–1.07)	32	1.10 (0.90–1.53)	0.0229	19	80.0 (70.0–100.0)	32	87.0 (73.5–106.5)	0.4770	12	4.17 (3.62–4.55)	28	3.92 (3.49–4.70)	0.7455
HxH3	7	0.79 (0.77–0.88)	9	1.30 (1.09–1.83)	0.0002	7	261.0 (234.0–297.0)	9	215.0 (205.0–307.5)	0.6806	1	3.73	6	3.74 (3.47–4.23)	--
H3H3	4	0.45 (0.20–0.75)	0	--	--	4	498.5 (488.5–543.0)	0	--	--	2	5.16	0	--	--
K-W test *p*		0.0008		--			<0.0001		--			0.3450		--	

c.1418CC	45	1.03 (0.83–1.28)	99	1.24 (1.07–1.60)	0.0002	43	101.0 (80.0–122.0)	99	101.0 (82.0–160.0)	0.3928	43	4.27 (3.85–4.82)	87	3.82 (3.51–4.50)	0.0495
c.1418CT	10	1.05 (0.95–1.37)	43	1.29 (1.09–1.62)	0.0673	9	109.0 (93.0–175.5)	43	100.0 (82.0–115.0)	0.2818	8	4.00 (3.44–4.27)	33	3.80 (3.32–4.48)	0.8051
c.1418TT	1	1.23	10	1.29 (1.16–1.66)	--	1	81.0	10	92.5 (68.0–154.0)	--	1	2.47	9	3.00 (2.60–3.45)	--
K-W test *p*		0.6866		0.7600			0.4530		0.5174			0.1338		0.0032	

x ≠ 1 and 3. APC indicates activated protein C; M-W, Mann–Whitney U; K–W, Kruskal–Wallis; sEPCR, soluble endothelial protein C receptor; sTM, soluble thrombomodulin.

## Supplementary Materials

The following are available online at https://www.mdpi.com/1422-0067/21/16/5651/s1, Table S1: Differences in the clinical characteristics of the study subjects. The levels of each clinical variable registered were compared between groups using the Kruskal–Wallis test for continuous variables and the Chi-squared test with Holm correction for categorical variables. Results were considered statistically significant at *p* < 0.05. All statistical analyses were performed using R (version 3.5.1); Table S2: Levels of neutrophil activation markers, APC, sEPCR, and sTM in VTE patients according to cause, provoked or unprovoked DVT. Differences in every marker studied were assessed using the Wilcoxon–Mann–Whitney test. Results were considered statistically significant at *p* < 0.05. All statistical analyses were performed using R (version 3.5.1); Table S3: Levels of neutrophil activation markers in lower extremity VTE, SVT, and all patients according to VTE recurrences. Differences in every marker studied were assessed using the Wilcoxon–Mann–Whitney test. Results were considered statistically significant at *p* < 0.05. All statistical analyses were performed using R (version 3.5.1); Table S4. Multivariable logistic regression model to assess the variation of the thrombotic risk according to the concentration of cfDNA. Covariates were age, sex, and presence of the thrombophilic risk factors FVL and PT g.20210G>A. All statistical analyses were performed using R (version 3.5.1); Table S5. Multivariable logistic regression model to assess the variation of the thrombotic risk according to the concentration of myeloperoxidase. Covariates were age, sex, and presence of the thrombophilic risk factors FVL and PT g.20210G>A. All statistical analyses were performed using R (version 3.5.1); Table S6. Multivariable logistic regression model to assess the variation of the thrombotic risk according to the concentration of calprotectin. Covariates were age, sex, and presence of the thrombophilic risk factors FVL and PT g.20210G>A. All statistical analyses were performed using R (version 3.5.1); Table S7. Multivariable logistic regression model to assess the variation of the thrombotic risk according to the concentration of cfDNA, calprotectin, and myeloperoxidase. Covariates were age, sex, and presence of the thrombophilic risk factors FVL and PT g.20210G>A. All statistical analyses were performed using R (version 3.5.1).

## Abbreviations

APC	Activated Protein C
cfDNA	Cell-free DNA
DVT	Deep vein thrombosis
EPCR	Endothelial protein C receptor
H	Haplotype
MPO	Myeloperoxidase
NETs	Neutrophil extracellular traps
NLR	Neutrophil-to-lymphocyte ratio
PE	Pulmonary embolism
*PROCR*	Gene encoding for the endothelial protein C receptor
sEPCR	Soluble endothelial protein C receptor
sTM	Soluble thrombomodulin
SVT	Splanchnic vein thrombosis
*THBD*	Gene encoding for thrombomodulin
TM	Thrombomodulin
VTE	Venous thromboembolism
