# Supplementary material for: Increase of Neutrophil Activation Markers in Venous Thrombosis—Contribution of Circulating Activated Protein C
Source: Int J Mol Sci. 2020 Aug 6;21(16):5651. doi: 10.3390/ijms21165651 (PMC7460596; doi:10.3390/ijms21165651)
Supplement: Supplementary file 1 [file ijms-21-05651-s001.pdf]

Table S1. Differences in the clinical characteristics of the study subjects. The levels of each clinical variable registered were compared between groups using the Kruskal–Wallis test for continuous variables, and the Chi-squared test with Holm correction for categorical variables. Results were considered statistically significant at $p < 0.05$. All statistical analyses were performed using R (version 3.5.1).

Clinical characteristics	Patients			Controls (D)	A *vs.* B	A *vs.* D	B *vs.* D	C *vs.* D
	Lower extremity VTE (A)	SVT (B)	Total (C)					
**N (% of total)**	192 (68.6)	61 (21.8)	253	249	----	----	----	----
**Age, y**	45 (36, 55)	58 (46, 66)	47 (37, 60)	41 (32, 55)	**<0.001**	0.094	**<0.001**	**<0.001**
**Age at first onset, y**	42 (33, 51)	53 (38, 61)	42 (34, 53)	----	0.130	----	----	----
**Male sex, N (%)**	106 (55.2)	47 (77.0)	153 (60.5)	117 (47.0)	**<0.05**	0.182	**<0.001**	**<0.05**
**Recurrent thrombosis, N (%)**	57 (29.7)	6 (9.8)	63 (24.9)	----	**<0.01**	----	----	----
**Familial thrombosis, N (%)**	69 (35.9)	20 (32.8)	89 (35.2)	---	0.350	----	----	----
**Spontaneous thrombosis, N (%)**	48 (25.0)	3 (4.9)	51 (20.2)	----	**<0.001**	----	----	----
**Leukocytes, x 10^9/L**	6.4 (5.3, 7.4)	4.6 (3.6, 5.5)	5.9 (4.8, 7.1)	6.4 (5.5, 7.9)	**<0.001**	0.172	**<0.001**	**<0.001**
**Neutrophils, x 10^9/L**	3.5 (2.9, 4.3)	2.6 (2.1, 3.3)	3.2 (2.5, 4.1)	3.7 (3.0, 4.6)	**<0.001**	0.291	**<0.001**	**<0.001**
**Monocytes, x 10^9/L**	0.5 (0.4, 0.6)	0.5 (0.4, 0.7)	0.5 (0.4, 0.6)	0.5 (0.4, 0.6)	0.581	0.182	0.857	0.070
**Eosinophils, x 10^9/L**	0.15 (0.10, 0.22)	0.13 (0.08, 0.24)	0.15 (0.10, 0.23)	0.18 (0.10, 0.21)	>0.999	0.708	>0.999	0.530

**Basophils**, x 10^9/L	0.01 (0.00, 0.03)	0.01 (0.00, 0.02)	0.01 (0.00, 0.03)	0 (0.00, 0.02)	0.717	0.130	0.128	**<0.05**
**Lymphocytes, x 10^9/L**	1.90 (1.53, 2.34)	1.12 (0.78, 1.53)	1.71 (1.23, 2.20)	2.10 (1.71, 2.60)	**<0.001**	**<0.01**	**<0.001**	**<0.001**
**Platelets, x 10^9/L**	223 (188, 257)	97 (67, 153)	205 (143, 248)	238 (204, 276)	**<0.001**	**<0.05**	**<0.001**	**<0.001**
**Neutrophil-to-lymphocyte ratio**	1.81 (1.44, 2.35)	2.26 (1.64, 3.41)	1.90 (1.46, 2.52)	1.68 (1.33, 2.24)	**<0.01**	0.172	**<0.001**	**<0.01**
**FV Leiden, N (%)**								
**-/-**	166 (86.5)	59 (96.7)	225 (88.9)	244 (98.0)	0.862	**<0.001**	0.950	**<0.01**
**+/- & +/+**	25 & 1 (13.5)	2 & 0 (3.3)	27 & 1 (10.0)	4 & 1 (2.0)				
**PT g.20210G>A, N (%)**								
**GG**	176 (91.7)	57 (93.4)	233 (92.1)	236 (94.8)	>0.999	>0.999	>0.999	>0.999
**GA & AA**	16 & 0 (8.9)	4 & 0 (6.6)	20 & 0 (7.9)	13 & 0 (5.2)				

Continuous variables are displayed as median and interquartile range. Categorical variables are displayed as count and percentage. Significant differences are marked in bold.

Table S2. Levels of neutrophil activation markers, APC, sEPCR, and sTM in VTE patients according to cause, provoked or unprovoked DVT. Differences in every marker studied were assessed using the Wilcoxon–Mann–Whitney test. Results were considered statistically significant at $p < 0.05$. All statistical analyses were performed using R (version 3.5.1).

Marker	**Provoked DVT (N=88)**	**Unprovoked DVT (N=48)**	***P***
**cfDNA (ng/mL)**	1626.1 (1388.5, 1832.8)	1586.0 (1460.4, 1722.3)	0.591
**calprotectin (ng/mL)**	99.0 (67.3, 124.5)	118.0 (78.5, 160.0)	0.087
**myeloperoxidase (ng/mL)**	1432.5 (943.0, 2127.1)	1801.5 (960.1, 3052.0)	0.379
**APC (ng/mL)**	1.07 (0.86, 1.25)	1.06 (0.89, 1.21)	0.878
**sEPCR (ng/mL)**	102.5 (84.2, 203.8)	100 (83.1, 112.5)	0.405
**sTM (ng/mL)**	4.28 (3.78, 5.06)	4.39 (3.90, 4.78)	0.894

Continuous variables are displayed as median and interquartile range.

Table S3. Levels of neutrophil activation markers in lower extremity VTE, SVT, and all patients according to VTE recurrences. Differences in every marker studied were assessed using the Wilcoxon–Mann–Whitney test. Results were considered statistically significant at $p < 0.05$. All statistical analyses were performed using R (version 3.5.1).

Neutrophil activation marker	**Lower extremity VTE**		**SVT**		**All patients**		***P*** **A *vs.* B**	***P*** **C *vs.* D**	***P*** **E *vs.* F**
	**Recurrences (N=57) (A)**	**No recurrences (N=135) (B)**	**Recurrences (N=6) (C)**	**No recurrences (N=55) (D)**	**Recurrences (N=63) (E)**	**No recurrences (N=190) (F)**			
**cfDNA (ng/mL)**	1586 (1435, 1812)	1635 (1408, 1838)	1492 (1286, 1842)	1580 (1366, 1778)	1586 (1427, 1824)	1626 (1388, 1821)	0.812	0.701	0.631
**calprotectin (ng/mL)**	106 (77, 130)	114 (75, 156)	95 (67, 112)	92 (61, 165)	103 (73, 126)	112 (67, 158)	0.573	0.916	0.754
**myeloperoxidase (ng/mL)**	1751 (923, 2372)	1717 (1080, 2940)	1875 (1001, 1950)	1815 (1300, 3062)	1801 (939, 2317)	1740 (1110, 3000)	0.671	0.479	0.421

Continuous variables are displayed as median and interquartile range.

Table S4. Multivariable logistic regression model to assess the variation of the thrombotic risk according to the concentration of cfDNA. Covariates were age, sex, and presence of the thrombophilic risk factors FVL, and PT g.20210G>A. All statistical analyses were performed using R (version 3.5.1).

	Estimate	Std. Error	OR	95% CI
**Intercept**	-33.409	3.758	0	0
**log(cfDNA)**	4.475	0.508	87.792	32.771, 239.687
**Age**	0.024	0.008	1.024	1.009, 1.041
**Male sex**	-0.444	0.216	0.641	0.417, 0.969
**FVL**	1.471	0.502	4.352	1.662, 12.223
**PT g.20210G>A**	-0.004	0.418	0.996	0.443, 2.304
**WAIC**	458.279	16.499		

Std. Error, standard error; OR, Odds ratio; CI, confidence interval; FVL, Factor V Leiden; PT, prothrombin; WAIC, Watanabe-Akaike Information Criterion.

Table S5. Multivariable logistic regression model to assess the variation of the thrombotic risk according to the concentration of myeloperoxidase. Covariates were age, sex, and presence of the thrombophilic risk factors FVL, and PT g.20210G>A. All statistical analyses were performed using R (version 3.5.1).

	Estimate	Std. Error	OR	95% CI
**Intercept**	-4.332	1.203	0.013	0.001, 0.139
**log(MPO)**	0.474	0.154	1.606	1.198, 2.180
**Age**	0.024	0.009	1.025	1.008, 1.042
**Male sex**	-0.215	0.232	0.807	0.507, 1.254
**FVL**	1.421	0.497	4.141	1.648, 11.238
**PT g.20120G>A**	0.327	0.431	1.387	0.616, 3.286
**WAIC**	449.866	11.410		

Std. Error, standard error; OR, Odds ratio; CI, confidence interval; FVL, Factor V Leiden; MPO, myeloperoxidase; PT, prothrombin; WAIC, Watanabe-Akaike Information Criterion.

Table S6. Multivariable logistic regression model to assess the variation of the thrombotic risk according to the concentration of calprotectin. Covariates were age, sex, and presence of the thrombophilic risk factors FVL, and PT g.20210G>A. All statistical analyses were performed using R (version 3.5.1).

	Estimate	Std. Error	OR	95% CI
**Intercept**	-2.822	0.895	0.059	0.010, 0.337
**log(calprotectin)**	0.554	0.183	1.741	1.226, 2.493
**Age**	0.015	0.008	1.015	1.000, 1.031
**Male sex**	-0.399	0.214	0.671	0.440, 1.028
**FVL**	1.745	0.525	5.727	2.162, 16.642
**PT g.20210G>A**	0.447	0.425	1.564	0.679, 3.684
**WAIC**	490.326	11.779		

Std. Error, standard error; OR, Odds ratio; CI, confidence interval; FVL, Factor V Leiden; PT, prothrombin; WAIC, Watanabe-Akaike Information Criterion.

Table S7. Multivariable logistic regression model to assess the variation of the thrombotic risk according to the concentration of cfDNA, calprotectin, and myeloperoxidase. Covariates were age, sex, and presence of the thrombophilic risk factors FVL, and PT g.20210G>A. All statistical analyses were performed using R (version 3.5.1).

	Estimate	Std. Error	OR	95% CI
**Intercept**	-32.198	4.556	0	0
**log(cfDNA)**	3.765	0.590	43.162	13.858, 139.527
**log(calprotectin)**	0.287	0.228	1.333	0.862, 2.089
**log(MPO)**	0.379	0.181	1.461	1.025, 2.087
**Age**	0.021	0.011	1.021	1.000, 1.043
**Male sex**	-0.251	0.283	0.778	0.450, 1.358
**FVL**	1.515	0.526	4.548	1.737, 13.065
**PT g.20120G>A**	0.360	0.536	1.434	0.499, 4.255
**WAIC**	314.283	13.054		

Std. Error, standard error; OR, Odds ratio; CI, confidence interval; FVL, Factor V Leiden; MPO, myeloperoxidase; PT, prothrombin; WAIC, Watanabe-Akaike Information Criterion.
